# Urinary fistula-A continuing problem with changing trends

**DOI:** 10.4274/jtgga.2016.0211

**Published:** 2017-03-01

**Authors:** Sunesh Kumar, Richa Vatsa, Juhi Bharti, Kallol Kumar Roy, Jai Bhagwan Sharma, Neeta Singh, Jyoti Meena, Seema Singhal

**Affiliations:** 1 Department of Obstetrics and Gynecology, All India Institute of Medical Sciences, New Delhi, India

**Keywords:** Urinary fistula, vesicovaginal, obstructed labor, iatrogenic

## Abstract

**Objective:**

Urinary fistula is a distressing complication after difficult vaginal deliveries, obstetric, and gynecologic surgeries. The present study describes a single center’s experience in the management of urinary fistula at a tertiary care hospital. It was performed to analyze the etiology of genitourinary fistula, to assess the outcome after surgical repair, and to determine the changing trends in the etiology and management of urinary fistula.

**Material and Methods:**

This retrospective study was conducted over 5 years in the department of obstetrics and gynecology, All India Institute of Medical Sciences, New Delhi. Twenty patients who underwent surgical repair of urinary fistula were included in the study and analyzed for their etiology, presentation, site, size, previous failed repair, approach of surgical repair, and outcome. The findings of the present study were compared with a previous study at our center to determine the changing trends of urinary fistula.

**Results:**

The mean age of the study population was 37.05±8.08 years. The majority (65%) of the fistulae occurred following gynecologic surgeries, whereas 25% were due to obstructed labor, and 10% after cesarean section for other indications. The vaginal approach was used in all except one case of uterovesical fistula, which was repaired abdominally. The outcome was successful in 85% of cases. The success rate was similar in primary versus previous failed repair (p=0.270).

**Conclusion:**

The most common cause of urinary fistula in the present study was gynecologic surgery. The vaginal approach can be used even in cases with previous failed repairs with a high success rate.

## INTRODUCTION

Urinary fistula is one of the most distressing complications after difficult vaginal deliveries, obstetric, and gynecologic surgeries. It has great impact on social, psychological, and sexual life of affected patients. It is a very old entity, the earliest case was reported in 1923 in a mummified body that was dated 2050 BC (1). The reported incidence of vesicovaginal fistula (VVF) in developed countries is 0.3-2% (2). However, the exact incidence in the developing world is not known, probably due to the underreporting of cases (3). A study estimated the prevalence of VVF in the reproductive age group as 1.60 per 1000 women in South Asia (4). The burden can be estimated by the fact that according to an estimation in Ethiopia alone, 9000 women develop fistula annually, and only 1200 of those were being treated (5).

 In the past, the most common cause of VVF was prolonged and obstructed labor. Up to 90% of fistulae in developing countries were obstetric in origin (6), whereas almost 70% were due to gynecologic causes in developed countries such as the United States and United Kingdom (7). All this reflected poor access to skilled obstetric care in the majority of the population (8). In recent years, there has been a shift in etiology of urinary fistula. The trend has changed from obstetric to gynecologic causes, mainly due to the advancement in obstetric practices, increased institutional delivery, improved intrapartum care, and availability of emergency cesarean sections (CS) (9). In a recent metaanalysis, the gynecologic contribution was 81-91% (10). In a study, the incidence of VVF after abdominal hysterectomy for benign conditions and vaginal hysterectomies for prolapse was reported as 0.18% and 0.025%, respectively (11). Another study reported the incidence after total laparoscopic hysterectomy as 0.16% (12).

Repair of VVF can be achieved transvaginally or transabdominally. The choice of route depends on the characteristics of the fistula and the experience of the surgeon. The present study describes a single center’s experience in the management of urinary fistula at a tertiary care hospital. The objective of study was to analyze the etiology of genitourinary fistula, to assess the outcome after surgical repair, and also to determine changing trends in the etiology and management of urinary fistulae.

## MATERIAL AND METHODS

The study was conducted in the department of obstetrics and gynecology, All India Institute of Medical Sciences, New Delhi, over a period of 5 years (2011-2015). Twenty patients who underwent surgical repair of urinary fistula were included in the study after approval was obtained from the Ethics Committee of the institution. Informed written consent was given by all patients. The hospital records of these patients were reviewed. Patients with previous failed repair of fistula were also included. Detailed history was taken including age, parity, etiology of fistula, presentation, previous surgical repair and their outcome. All the patients were examined for site of fistula, number, size, fibrosis, and vaginal stenosis. A three-swab test was performed to clearly locate the fistula, with the patient in the lithotomy position. Three swabs were placed in the vagina, one above the other, followed by retrograde filling of the bladder with diluted and sterilized methylene blue by means of a per urethral catheter. Patients were then asked to walk around or perform the Valsalva manoeuvre. The swabs were then examined after 15 minutes to determine the site of the fistula. If the topmost swab was discolored, it indicated a VVF. Only wetting of the topmost swab without discoloration indicated a ureterovaginal fistula. Discoloration of only the lowest swab was suggestive of a low urethral fistula or back flow from the introitus. Cystoscopy was performed in all patients to see the number and location of fistula in relation to the trigone of the bladder. A urine culture and sensitivity was performed using a sterile speculum in all patients prior to surgery.

The fistulae were repaired via the transvaginal route using the flap-splitting technique in all cases but one case of uterovesical fistula, which was repaired transabdominally. In the flap-splitting technique, the fistula was identified and a pediatric Foley catheter was inserted through the fistulous opening. Traction on the catheter made the fistula more accessible and a circumferential incision was then made around the fistulous opening. A vaginal mucosal flap was lifted away from bladder to an extent that allowed tension-free closure of the defect. The bladder was closed in two layers using a delayed absorbable suture (Polyglactin 3-0). In the first layer, the bladder mucosa was closed with interrupted sutures. The second layer included bladder muscularis and perivesical fascia in an interrupted fashion and hemostasis ensured. After this, bladder integrity was checked using diluted methylene blue dye. The vaginal mucosa was then closed over it in a running interlocking manner with Polyglactin 2-0. Continuous bladder drainage was ensured in all patients for 14 to 21 days using a per urethral catheter. Broad-spectrum antibiotics were given and patients were followed up 1 month, 2 months, and 6 months after surgery. The success of the repair was assessed at the end of 6 months by examination and a repeat three-swab test. The findings of the present study were compared with a previous study at our center to determine changing trends of urinary fistula in a developing country such as India (13).

Statistical analysis was performed using Statistical Package for the Social Sciences software, International Business Management version 20.0. (Armonk, New York, USA). Descriptive statistics such as mean, standard deviation, range and median values were calculated for continuous variables such as age of patients and duration of symptoms. Frequencies of outcomes across the categories are represented as frequency and percentage values. To compare the frequency of occurrences of outcomes across categories, Chi-square/Fisher’s exact tests were used as appropriate. For all tests, a two-tailed probability of p<0.05 was considered statistically significant.

## RESULTS

The mean age of the study population was 37.05±8.08 years (range, 20-50 years). Patients presented with symptoms for a median duration of 8 months. Table 1 shows the antecedent events that led to fistula in our cases. The majority (65%) were due to gynecologic causes, most of which occurred after abdominal hysterectomy (45%). All except one fistulae in our study were vesicovaginal, the remainder was a uterovesical fistula that occurred following CS. There were two cases of fistulae following CSs performed for indications other than obstructed labor, one was VVF following CS performed for a morbidly adherent placenta, and the other was a uterovesical fistula where there were dense adhesions of the bladder involving the uterus due to previous cesarean section. Based on the cystoscopy findings, the majority (75%) of the fistulae was supratrigonal. This was followed by fistula on the bladder neck in 15%, and trigonal in 10%.

We also classified fistula as simple and complicated based on characteristics of fistula, such as number, site, size, urethral involvement, scarring of tissue, and number of previous repairs (14). Simple fistulae were present in 11 (55%) women, and 9 (45%) had complicated fistulae. Thirteen (65%) patients underwent primary repair, and 7 (35%) had at least one previous failed repair elsewhere. All the repairs in our study except one were performed vaginally, the remainder was a uterovesical fistula, which was repaired abdominally. At the 6^th^ month follow-up, 17 patients had a successful result (85%) and 3 (15%) had failed repairs. The outcome of surgery was compared in simple versus complicated group, and also regarding the number of previous repairs (Table 2). There was no statistically significant difference between the success rates of simple and complicated fistulae, or between primary and previous repair.

## DISCUSSION

The mean age of the patients in the present study was 37.05 ±8.08 years. In recent studies by Wadie and Kamal (15) in 2011 and Karateke et al. in 2010 (16), the median age at presentation was >35 years, which is similar to the present study. Wall et al. (17) in 2004 reported a higher incidence of VVF in a younger population; the mean age was 27 years (range, 13-20 years). The difference in age of presentation over the years is due to different antecedent events leading to fistula. Obstetric fistula used to be a major cause of genitourinary fistula. Wall et al. (17) reported almost 96.5% cases of urinary fistula due to obstetric causes in developing parts of the world like sub-Saharan African nations, due to poor obstetric care. In the previous study from our center, 73.9% of fistulae were obstetric related (12). This is in contrast to the present study, where the majority of fistulae were gynaecologic in origin (Table 3). Similar results were reported by another study where hysterectomy accounted for 91% of fistulae (83% abdominal, 9% vaginal surgeries) (2). In our study too, abdominal hysterectomy accounted for most fistulae. This is partly due to improved obstetric care services, and the increasing number of gynecologic surgeries. Obstructed labor as a cause of fistula is showing a declining trend due to an increase in delivery rates by skilled birth attendants and the availability of round-the-clock emergency cesarean services. During abdominal hysterectomy, VVF can result due to direct injuries resulting from sharp dissection of the bladder in the wrong plane, especially in cases of previous pelvic surgeries, endometriosis or malignancies. It could also be due to iatrogenic injury by inadvertent sutures in the bladder wall or thermal cautery burn leading to necrosis of the wall.

There is a long gap between the appearance of symptoms and presentation to health care facility in these women. The median duration of symptoms in a study was 11.5 months (range, 3-228 months) (15). In the present study, this duration was 8 months (range, 1-228 months). Even today, there is a delay in seeking health care in these women. This condition is socially debilitating and these women are usually neglected in their families, which could be the reason for this delay. A difference in opinion exists regarding the route of repair. The choice of route of repair depends on the nature of the fistula. However, the most important factor is the choice of surgeons and their experience. Eilber et al. (2) used the vaginal approach in most cases because the abdominal approach was associated with greater morbidity such as greater blood loss, longer hospital stay, more pain, and cost. Nevertheless, they considered that the surgeon’s choice was the most important. In another study, the same authors used the abdominal approach in 68% cases, vaginal in 25%, and a combined approach in 7% (15). We used the vaginal approach in all patients except one who had a uterovesical fistula. Even patients with previous failed repairs underwent vaginal access procedures. The abdominal approach has greater blood loss, prolonged hospitalization, and increased postoperative pain, but the success rates are similar (18, 19).

We achieved an overall success rate of 85% in the present study. Complicated fistulae were also managed using the vaginal approach. The outcome of surgery in relation to fistula type and previous failed repair is shown in Table 3. Another study by Wadie and Kamal (15) also reported a cure rate of 91% with abdominal access and 70% with the vaginal approach, the reason for choosing a particular route was surgeon’s preference. Kapoor et al. (20) achieved very a high success rate of 94.2% in their study, but they used the abdominal approach for complex fistulas. The findings of the present study were compared with a previous study from the same center that was conducted between 1999 and 2004 (12). The important points are summarized in Table 3. This table highlights the change in etiology of fistula from obstetric to gynaecologic causes. In both the studies, the preferred approach was vaginal and the success of repair was almost the same. The limitations of our study are its small sample size and retrospective nature.

## CONCLUSION

The causes of urinary fistula have changed significantly over the last few decades, even in developing countries such as India. The trend is shifting from obstetric to gynecologic causes. The vaginal approach has a high success rate and less morbidity, even in patients with previous failed repairs. Emphasis should be laid on preventing this dehumanizing condition.

## Figures and Tables

**Table 1:**
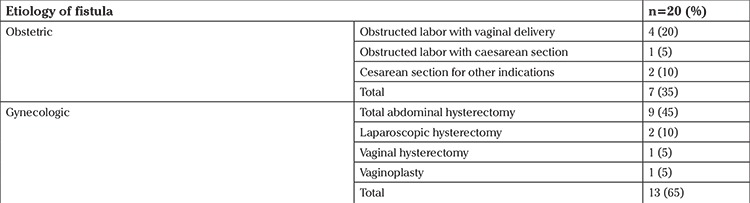
Etiology of fistula

**Table 2:**
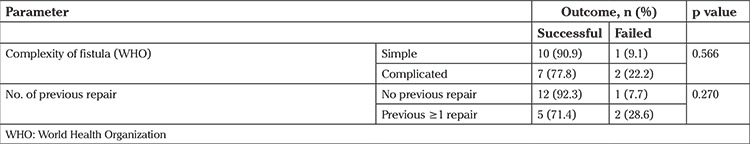
Outcome of fistula repair based on type of fistula and number of previous repair

**Table 3:**
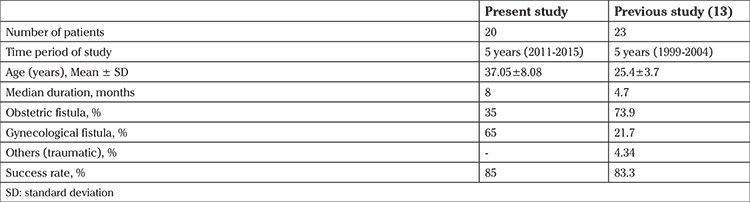
Comparison between present study and a previous study from same center
